# A blood-based composite panel that screens Alzheimer’s disease

**DOI:** 10.1186/s40364-023-00485-6

**Published:** 2023-05-16

**Authors:** Yan Wang, Ying Li, Yan Li, Tingting Li, Qi Wang, Qigeng Wang, Shuman Cao, Fangyu Li, Jianping Jia

**Affiliations:** 1 Innovation Center for Neurological Disorders and Department of Neurology, Xuanwu Hospital, Capital Medical University, National Clinical Research Center for Geriatric Diseases, Beijing, China; 2 Beijing Key Laboratory of Geriatric Cognitive Disorders, Beijing, China; 3 Clinical Center for Neurodegenerative Disease and Memory Impairment, Capital Medical University, Beijing, China; 4 Center of Alzheimer’s Disease, Beijing Institute of Brain Disorders, Collaborative Innovation Center for Brain Disorders, Capital Medical University, Beijing, China; 5 Key Laboratory of Neurodegenerative Diseases, Ministry of Education, Beijing, China

**Keywords:** Alzheimer’s disease, Mild cognitive impairment, Blood, Biomarker

## Abstract

**Background:**

Blood tests would be much easier to implement in the clinical diagnosis of Alzheimer’s disease (AD) as minimally invasive measurements. Multiple inspection technologies promoted AD-associated blood biomarkers’ exploration. However, there was a lack of further screening and validation for these explored blood-based biomarkers. We selected four potential biomarkers to explore their plasma levels in AD and amnestic mild cognitive impairment (aMCI) and developed a composite panel for AD and aMCI screening.

**Method:**

The plasma concentrations of soluble low-density lipoprotein receptor-associated protein 1 (sLRP1), Gelsolin (GSN), Kallikrein 4 (KLK4) and Caspase 3 were measured in the discovery and validation cohort. The receiver operating characteristic (ROC) curve was generated to assess the classification panel with the area under the curve (AUC).

**Results:**

A total of 233 participants (26 CN, 27 aMCI, and 26 AD in the discovery cohort, and 51 CN, 50 aMCI, and 53 AD in the validation cohort) with complete data were included in the study. The plasma concentrations of sLRP1 and Caspase 3 were significantly decreased in AD and aMCI when compared with those in the CN group. Compared with the CN group, the concentrations of KLK4 and GSN were increased in AD, but not in MCI. Interestingly, one of four proteins, sLRP1 in plasma level was higher in Apolipoprotein E (APOE) *ε4* non-carriers than that in APOE *ε4* carriers, especially among CN and MCI. No significant difference was found between females and males in the plasma levels of four proteins. The composite panel is based on four blood biomarkers accurately classifying AD from CN (AUC = 0.903–0.928), and MCI from CN (AUC = 0.846–0.865). Moreover, dynamic changes in the plasma levels of four proteins exhibited a significant correlation with cognitive assessment.

**Conclusions:**

Altogether, these findings indicate that the plasma levels of sLRP1, KLK4, GSN and Caspase 3 changed with the progression of AD. And their combination could be used to develop a panel for classifying AD and aMCI with high accuracy, which would provide an alternative approach for developing a blood-based test for AD and aMCI screening.

**Supplementary Information:**

The online version contains supplementary material available at 10.1186/s40364-023-00485-6.

## Background

Alzheimer’s disease (AD) is the leading cause of dementia, which is a dramatically increasing global health challenge [1]. From the national cross-sectional study in China, the prevalence of AD is 3.9%, with an estimated 9.83 million people aged 60 years or older. At the pre-dementia stage, the prevalence of mild cognitive impairment (MCI) was at 15.5%, with an estimated 38.77 million [2]. The diagnosis of AD and MCI is important to the prevention and treatment of this disease.

Thanks to plenty of clinical cohort studies, multiple biomarkers are presented and classified by diagnostic criteria and research, which provide a powerful tool to help clinicians with the diagnosis of AD and MCI [3]. In the ATN framework, Positron emission tomography (PET) imaging for Aβ, tau, and neuronal injury, and total tau, phosphorylated tau, and Aβ42/ Aβ40 in cerebrospinal fluid (CSF) is recommended [3]. However, the expensive costs of PET and the invasive procedure for CSF collection are greatly restricting their applications in AD and MCI screening. Instead, the blood-based test is an alternative, less-invasive, potential tool for the diagnosis of AD and MCI which was discovered and developed by an increasing body of research [4].

AD is a complex and continuous disease, that is involving multiple biological processes [5, 6]. During the progression of the disease, peripheral biological processes are also changed, which are possibly involved in pathological changes in the brain, such as inflammation, dysfunctional proteostasis, and hepatorenal metabolism [7–9]. Models combining biomarkers that represent multiple aspects of AD brain pathology may improve the prediction of AD dementia. Although it was a rapid increase in the number of potential blood-based biomarkers for diagnosis and monitoring of AD by proteomic technology [10–12], these explored proteins were lack of repeated validation and development of an effective diagnostic model for AD.

In a plasma proteomic profiling, a 19-protein panel was established for AD [10]. Six of these proteins were exhibiting consistent and significant alterations in patients with AD in discovery and validation [10]. Kallikrein 4 (KLK4) and Caspase 3 with high performance in the above study have the potential to be applied in the [10]. Given the typical pathological features in AD, we’re trying to search the potential biomarkers which could have the amyloid metabolism function [6]. The soluble low-density lipoprotein receptor-associated protein 1 (sLRP1) was a major peripheral transporter of amyloid beta, and Gelsolin(GSN) was also reported as an anti-amyloidogenic protein [13, 14]. Thus, we conducted the AD and MCI cohorts to measure the levels of the four proteins in peripheral blood, explore their relationships with the disease progression of AD, and build a simplified panel for AD screening.

In this study, we selected four potential biomarkers and measured their plasma levels in the discovery and validation cohort including AD, amnestic MCI (aMCI), and CN. Furthermore, the accuracy of the four biomarkers and their composite on the classification for AD and aMCI were estimated. Finally, we developed a composite panel based on the quantification of four biomarkers in plasma with high accuracy for classifying AD and aMCI. In addition, we found that these plasma proteins were remarkably correlated with cognitive and functional assessment among CN, aMCI, and AD. Thus, we established a four-blood-based protein panel for AD and aMCI, which provides an alternative approach for developing a blood-based test for AD and aMCI screening.

## Method

### Subject recruitment and clinical samples

The discovery cohort comprised 79 individuals, including 26 patients with AD, 27 patients with aMCI, and 26 normal cognitions (CN), who visited Xuanwu Hospital, Capital Medical University from January 2016 to December 2018. The validation cohort comprised 154 individuals, including 53 patients with AD, 50 patients with aMCI and 51 NCs, who visited other centers in Hebei, Shandong, Inner Mongolia, Guangxi and Jilin provinces, from June 2016 to January 2019. All participants underwent medical history assessment, clinical assessment, and cognitive and functional assessment using the Montreal Cognitive Assessment (MoCA). Diagnoses of probable AD were according to the 2011 criteria of the National Institute on Aging and Alzheimer’s Association (NIA-AA) [15]. Patients with aMCI were diagnosed based on the published criteria [16]. Participants with a psychiatric disorder or any significant neurological disease other than AD were excluded. The individuals’ information of age, sex, and years of education were recorded, and blood samples were collected at the visited center. All participants or their legal guardians provided written informed consent. This study was approved by the Institutional Review Board of Xuanwu Hospital, Capital Medical University.

### Biomarker measurements

The collected whole-blood samples were immediately processed at the local center. The plasma was obtained by centrifuging at 4200 × g for 10 min at room temperature, then kept in aliquots at − 80 °C until further testing. The enzyme-linked immunosorbent assay (ELISA) was employed to measure these biomarkers according to the kit manufacturer’s specifications. The ELISA kits used in this research are listed in Additional file 1: Table S1. Briefly, the samples and a series of standards were incubated with the capture antibody coated on microtiter wells for one or two hours. Then the liquid was removed, and the biotinylated detection antibody was added to be incubated for one hour. After washing three times, the avidin-peroxidase conjugated complex was added, which was continued to be incubated for one hour. After washing five times, TMB substrate was used to develop the signal which was stopped by sulfuric acid in 10–30 min. Then, the absorbance was read at 450 nm within 5 min. All measurements were performed blindly.

### Statistical analyses

Differences in demographic and clinical data and biomarker levels were tested with Chi-square and Mann-Whitney tests. The performance of the individual markers and the models was evaluated by using standard measurements of the area under the receiver operating characteristic (ROC) curve (AUC). Confidence intervals for the diagnostic parameters were calculated using the MedCalc application version 19.7.2 (MedCalc Software). All other analyses were performed with SPSS version 20 (IBM). The ROC curve was generated using GraphPad Prism version 8.0 (GraphPad Software). All other statistical plots were generated using GraphPad Software. Two-sided P values less than 0.05 were considered statistically significant.

## Results

### Participant characteristics

The demographic and clinical characteristics of discovery and validation cohorts are summarized in Table 1. In the discovery stage, aMCI patients were on average 67.03 years of age, the patients with AD were on average 66.42 years of age, and CN was on average 68.42 years of age The three groups did not differ significantly across demographic characteristics (Table 1), being comparable in mean age and sex. In the validation stage, there were also no differences in the ages and sex among AD, aMCI, and CN. While the education years were significantly higher among CN than that among AD and MCI in both discovery and validation cohorts. So it is with the percentages of APOE *ε4* as well. The scores of MoCA were significantly different (*P < 0.01*) between AD patients and CN, AD and aMCI patients, and aMCI patients and CN.

**Table 1:** Characteristics of participants in this study

Characteristic	Discovery			Validation		
	CN (n = 26)	MCI (n = 27)	AD (n = 26)	CN (n = 51)	MCI (n = 50)	AD (n = 53)
Age, mean(SD),y	68.42(8.45)	67.03(5.53)	66.42(5.91)	65.10(5.90)	66.22(5.70)	65.32(5.78)
Female, No.(%)	18(69.23)	13(48.15)	16(61.54)	33(64.70)	23(46.00)	29(54.72)
APOE *ε4* positive, No. (%)	4(15.38)	12(44.44)*	14(53.85)*	6(11.76)	20(40.00)*	27(50.94)*
MMSE score, mean (SD)	-	23.67(1.94)	14.88(5.19)☨	-	24.48(2.61)	14.88(5.19)☨
MoCA score, mean (SD)	27.85(0.88)	19.33(1.84)*	11.04(4.82)*☨	27.37(0.85)	19.08(3.34)*	9.58(5.80)*☨
Education, mean(SD),y	14.65(2.94)	10.07(3.86)*	8.58(4.07)*	13.65(2.57)	11.18(3.57)*	8.77(4.64)*☨

*P < 0.05 compared to controls. ☨*P < 0.05* compared to aMCI

### Plasma levels of four potential biomarkers among AD, aMCI, and CN

**Fig. 1:**
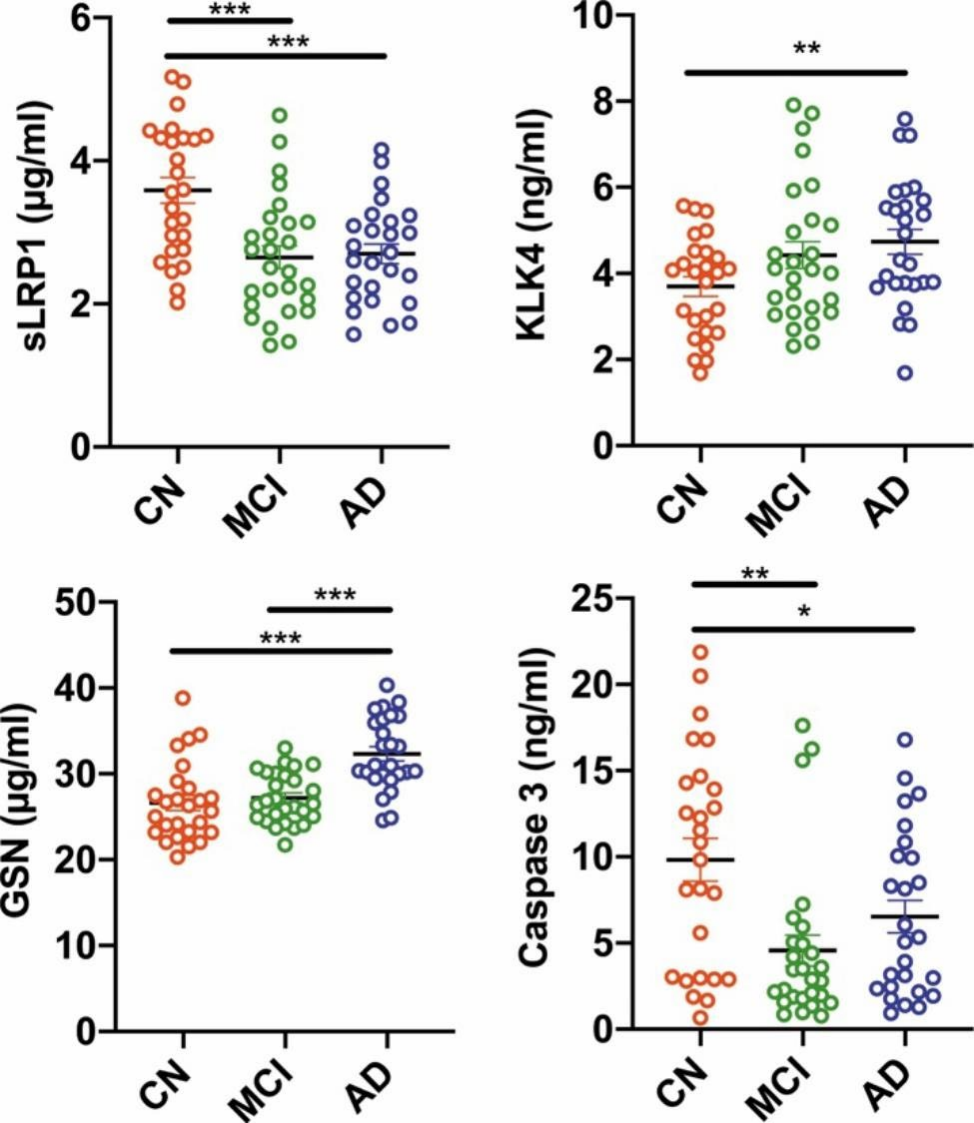
Individual plasma level of four biomarkers in a discovery cohort. A-D show levels of sLRP1, KLK4, GSN and Caspase 3. *P < 0.05, **P < 0.01, ***P < 0.001

As shown in Fig. 1, the plasma level of KLK4, and GSN in the AD group (4.74 ± 1.47 ng/ml, and 32.33 ± 4.23 µg/ml, *P < 0.01*) were significantly higher than those in the control group (3.70 ± 1.15 ng/ml and 26.62 ± 4.57 µg/ml,). There were no significant differences between aMCI and CN groups in plasma levels of KLK4, and GSN. While the concentrations of sLRP1 and Caspase 3 were significantly lower in the AD group (2.70 ± 0.70 µg/ml, and 6.53 ± 4.79 ng/ml, *P < 0.05*), when compared with these in the CN group (3.59 ± 0.92 µg/ml, and 9.83 ± 6.31 ng/ml). Furthermore, the concentrations of sLRP1 and Caspase 3 in the aMCI group (2.65 ± 0.83 µg/ml, and 4.56 ± 4.64 ng/ml, *P < 0.01*) were significantly lower than those in the control group. It was also found that there were significant differences between the aMCI and AD groups in plasma levels of GSN (aMCI 27.21 ± 2.93 µg/ml versus AD 32.33 ± 4.23 µg/ml, *P < 0.001*). These data hinted that four plasma proteins could be potentially used for distinguishing AD and aMCI from CN.

**Fig. 2:**
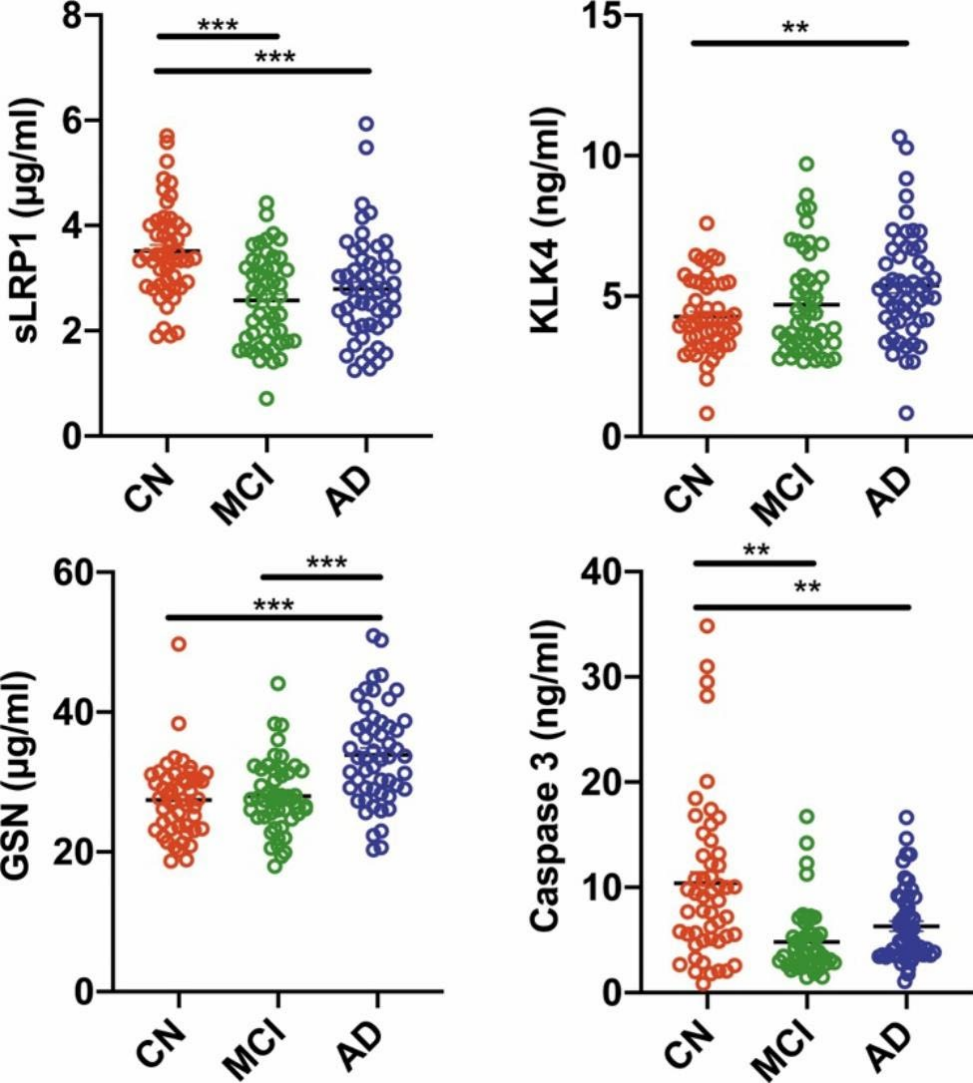
Validation of Individual plasma levels of four biomarkers in an independent cohort. A-D show levels of sLRP1, KLK4, GSN and Caspase 3. *P < 0.05, **P < 0.01, ***P < 0.001

Next, to validate whether the plasma-based biomarker panel could be used for high performance in AD and aMCI screening, we measured the plasma concentrations of four proteins in the validation cohort (Fig. 2). Compared to the four plasma proteins in the CN group, the plasma level of KLK4, and GSN (4.27 ± 1.31 ng/ml, 33.87 ± 7.08 µg/ml, respectively, *P < 0.001*) were significantly increased, and the plasma level of sLRP1 and Caspase 3 (2.79 ± 0.98 µg/ml, and 6.30 ± 3.50 ng/ml, *P < 0.01* or *0.001)* were significantly decreased in the AD group. There were no significant differences in plasma levels of KLK4 and GSN between the aMCI and CN groups. While the plasma concentrations of sLRP1 and Caspase 3 in the aMCI group (2.58 ± 0.86 µg/ml, and 4.81 ± 3.15 ng/ml, *P* < 0.01 or 0.001) were significantly lower than those in the CN group(3.51 ± 0.88 µg/ml, and 10.38 ± 7.74 ng/ml). Furthermore, there were also significant differences in plasma levels of GSN (aMCI 27.97 ± 5.13 µg/ml versus AD 33.87 ± 7.08 µg/ml, *P < 0.001*) between the MCI and AD groups. These data indicated that four protein biomarkers in plasma sufficiently distinguished AD patients from controls and aMCI patients from controls.

As reported, APOE *ε4* was one of the most risk genes in the development of AD [17]. We compared the plasma level of four proteins between APOE *ε4* carriers and non-carriers (Additional file 1: Fig. S1). The plasma level of sLRP1 was higher in APOE *ε4* non-carriers than that in APOE *ε4* carriers, especially among CN and aMCI groups. No significant difference was found in plasma levels of KLK4, GSN, and Caspase 3 between APOE *ε4* carriers and non-carriers. Since women were also one of the risk factors for AD [2], whether there were sex differences in plasma concentrations of four proteins was explored in combined cohorts (Additional file 1: Fig. S2). However, no significant difference was found in the plasma levels of four proteins between females and males. It follows that the plasma level of sLRP1 was possibly related to the APOE genotype, and plasma levels of four proteins were not related to sex.

### A blood-based biomarker panel accurately screens AD and aMCI

**Fig. 3:**
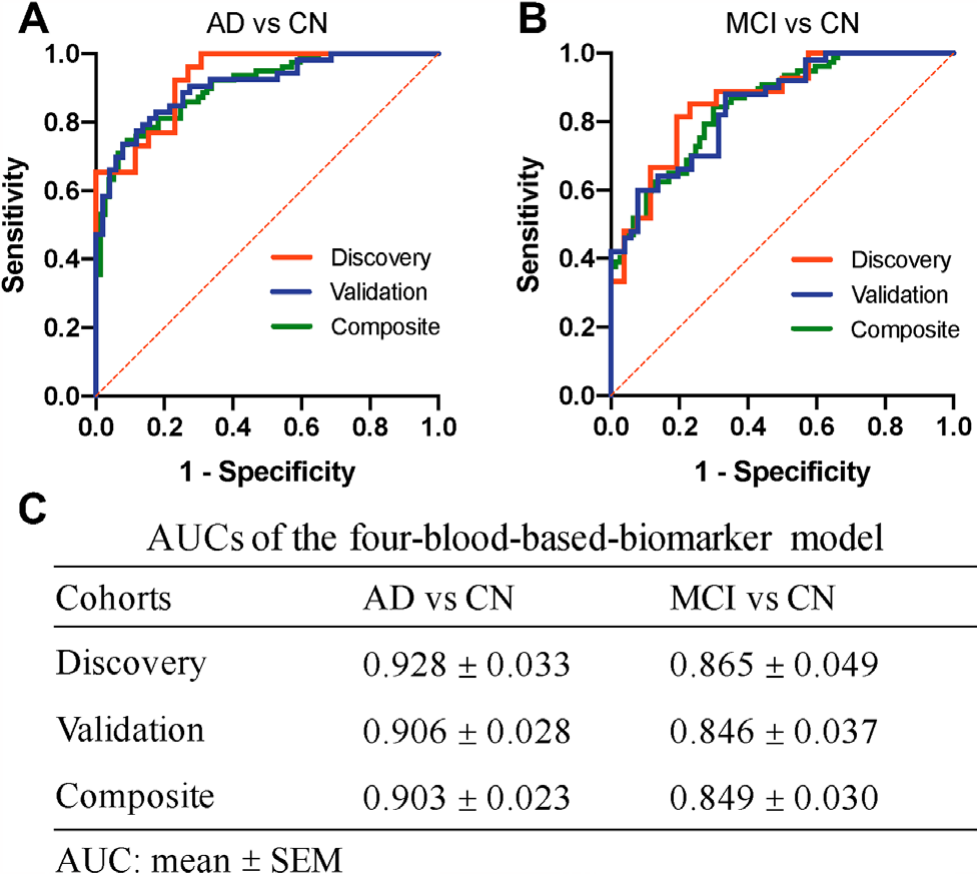
High performance of a four-blood-based biomarkers panel for AD and MCI classification. A, Receiver operating characteristic (ROC) curves showing the performance of the panel for AD classification in discovery (red), validation (blue), and combined (green) cohorts. B, ROC analyses for MCI classification in discovery (red), validation (blue), and combined (green) cohorts. C, Areas under the ROC curves (AUCs) of the four-blood-based biomarker panel for AD and MCI classification in discovery, validation, and combined cohorts. Data are mean ± SEM

Given that four protein biomarkers mentioned above were consistently altered in AD or aMCI plasma in the discovery and validation cohort, we examined whether they could also be used to classify AD patients from controls, and aMCI patients from controls by calculating the ROCs of KLK4, GSN, sLRP1 and Caspase 3 and their combination. The composite biomarkers panel was more powerful than any one biomarker for the classification of AD and aMCI (Additional file 1: Fig. S3). In the discovery cohort (Fig. 3), the composite panel of plasma biomarkers showed significant AUCs in the comparisons of AD/ CN (0.928, 95% CI: 0.863 to 0.993, *P < 0.001*), and aMCI/ CN(0.865, 95% CI: 0.769 to 0.961, *P < 0.001*). In the validation cohort (Fig. 3), analysis of the ROCs revealed similar results concerning AUCs (0.906 in the comparisons of AD/controls, and 0.846 in the comparisons of aMCI/controls). In combined cohorts, the composite panel based on four blood biomarkers accurately classified AD from controls (AUC = 0.903), and classified aMCI from controls (AUC = 0.849). Therefore, these results collectively demonstrated that the four-blood-based proteins panel had high accuracy for classifying AD and aMCI, providing an alternative approach for developing a blood-based test for AD and aMCI screening.

### Cognition-dependent dysregulation of the four plasma proteins in AD and aMCI

**Fig. 4:**
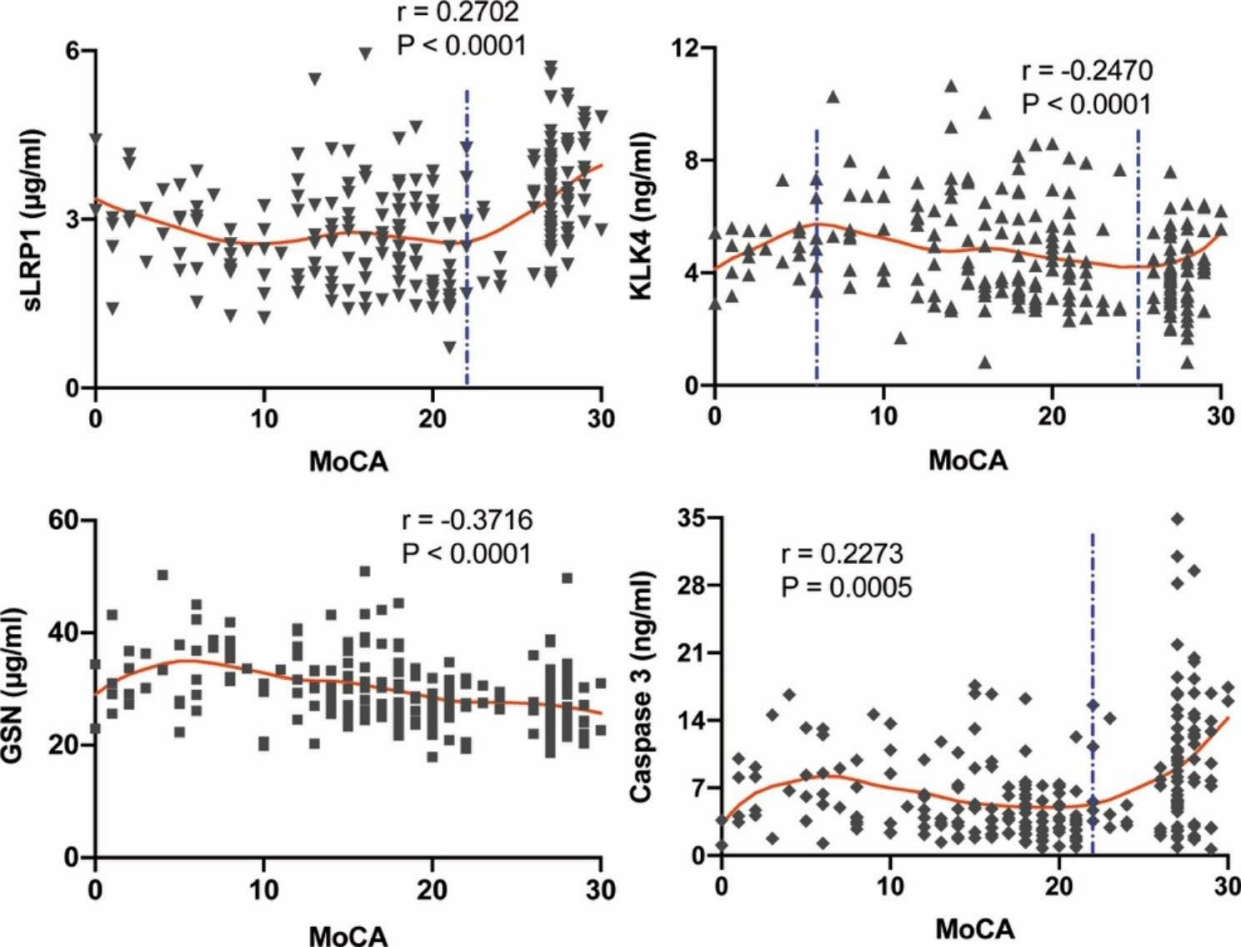
Associations between individual plasma level of four biomarkers and cognitive performance. A-D shows correlations between the plasma levels of sLRP1, KLK4, GSN, and Caspase 3 and MoCA scores. Red splines represent the locally weighted scatterplot smoothing (LOWESS) fit lines of corresponding biomarkers, and blue vertical dashed lines indicate the inflection points. *r*^2^, Pearson’s correlation coefficient

The progression of AD is continuous which could be marked by cognitive performance. We performed the correlation analysis between MoCA scores and the levels of the four plasma proteins in our cohort (Fig. 4). The results showed significant correlations between MoCA scores and GSN(r = -0.3716, *P < 0.0001*). KLK4 was dysregulated in the early stages of AD (MoCA > 25), increased as the disease progresses (MoCA: 6–25), then gradually fall (MoCA < 5). The plasma levels of sLRP1 and Caspase 3 were decreased at the early stages of AD (MoCA > 22) and continued at a low level throughout the disease’s progression. These results suggested that the changes in four plasma proteins were associated with cognitive changes in aMCI and AD. Certain plasma proteins are associated with corresponding biological processes. Therefore, the four identified proteins can be not only used as biomarkers to distinguish patients with AD or aMCI from CN, but serve as a biological scale for AD progression.

## Discussion

Blood-based tests are more accessible and inexpensive methods, which are needed for widespread applicability in clinical trials as well as for future implementation in routine clinical care [18]. In this study, we demonstrated that the concentrations of KLK4, GSN, Caspase 3, and sLRP1 in plasma were significantly different in AD, aMCI, and CN groups. Accordingly, a novel composite diagnostic panel based on four plasma biomarkers was developed, which could accurately distinguish patients with AD or aMCI from controls. Furthermore, we found that the plasma levels of these proteins were correlated with cognitive function assessment. We provide an innovative option for classifying AD and aMCI, and monitoring the progression of the disease.

In the novel diagnostic penal, sLRP1 was a key factor. As reported, sLRP1 is the major transport protein for peripheral Aβ, playing a role in clearing build-ups of the Aβ peptide [14]. In addition, LRP1 is a master regulator of tau uptake and spread [19]. Research indicated a deficient sLRP-Aβ binding might precede the increase in the tau/Aβ42 CSF ratio and global cognitive decline in MCI, and therefore it is an early biomarker of AD [20]. sLRP1 concentrations have been lower in patients with AD, and also could be used to distinguish AD from other types of dementia [21]. In our cohort, we found the plasma level of sLRP1 was remarkably decreased in aMCI and persistent at the low level in AD patients. It may indicate that a low concentration of sLRP1 decreases binding affinity for Aβ, resulting in elevations in free Aβ in plasma which may then lead to increased transport into the brain via RAGE [22]. And moreover, it was reported that APOE4 exacerbates Aβ pathology through a mechanism depending on neuronal LRP1 [23]. We also found there was a difference in the plasma level of sLRP1 depending on the individual APOE *ε4* genotype among CN and aMCI. Therefore, it would be interesting to explore that reduced sLRP1 in plasma may be a specific biochemical sign, and the interaction mechanism between sLRP1 and APOE at the preclinical AD stage.

Gelsolin(GSN) was included in our study via literature searches. GSN is a multifunctional actin-binding protein, which could be secreted in blood/cerebrospinal fluid implicating an actin scavenging system, presentation of lysophosphatidic acid and inflammatory mediators to receptors, and substrate function for matrix-modulating enzymes [24]. In AD, GSN as an anti-amyloidogenic protein, could bind and sequester Aβ, inhibit its fibrosis, and degrade formed fibres [13, 25, 26]. Some changes of gelsolin in blood or CSF were reported in AD progression, which contributes to its implication in AD diagnosis, while the plasma GSN levels were inconsistent in previous studies [27]. In our cohort, the plasma level of GSN was significantly increased in AD patients, but not in patients with aMCI. In a recent study, they also found an increase in plasma GSN in AD patients and they speculated GSN may function in the compensatory mechanism of Aβ pathology [27]. As the plasma GSN level was accompanied by changes in cognitive function, the GSN in plasma may be a potential biomarker for AD diagnosis.

The plasma KLK4 was recently identified as a hub protein associated with AD in a large-scale plasma proteomic profiling [10]. KLK4 is involved in the degradation of extracellular matrix proteins [28], and its role in AD was not reported by any other cohort except the above one [10]. In our study, the change in the plasma level of KLK4 was first measured in both aMCI and AD. And we found KLK4 was significantly increased in AD but not aMCI. And the concentration of KLK4 in plasma was dysregulated with cognitive function assessment(e.g. MoCA). Kallikrein-related peptidases (KLKs) are a subgroup of serine proteases, enzymes capable of cleaving peptide bonds in proteins, which are responsible for the coordination of various physiological functions. Although research on KLK4 in plasma was rarely in AD cohorts, other members of KLKs (e.g. KLK6, KLK7, and KLK8) had been studied in the diagnosis and pathogenic mechanism of AD [29–31]. It was reported that KLK4-7 together with other proteases may lead to the modulation of diverse signaling pathways accompanied by complex interactions [32]. Therefore, KLK4 might be involving an unknown mechanism in AD that needs to be further studied.

The Caspase 3 activity has been reported with marked increases in peripheral blood lymphocytes of AD, the concentration of Caspase 3 in plasma, however, was little reported among patients with AD. In our study, detectable Caspase 3 in plasma was significantly lower in aMCI and AD than that in CN, consistent with that in one plasma proteomic study among AD and CN. Based on the genetic and clinical data of peripheral blood RNA in GSE63060 and GSE63061, the expression levels of Caspase 3 in the peripheral blood were significantly decreased in AD and MCI [33], which was consistent with plasma protein levels of Caspase 3 in our cohort. Furthermore, we found the relationship between the plasma concentration of Caspase 3 and MoCA through the analysis of three groups, which were dysregulated in the early stage of AD. Thus, Caspase 3 could also be served as a potential candidate for incipient AD.

The composite biomarker panels are the concurrent use of multiple biomarkers to determine the status of the disease, which are widely used to predict cardiovascular diseases and aging [34, 35]. In the pathogenesis of AD, emerging evidence also showed multiplex pathways were associated with AD, such as inflammation, neurogenesis, metabolism, angiogenesis, etc. The composite biomarker panels could capture a broader spectrum of peripheral biological processes [10]. Recently, a panel of 19 proteins was established and classified AD with high accuracy [10]. In our study, a four-blood-based protein panel was developed, which could accurately classify AD or aMCI from controls. Although the accuracy of our panel was slightly lower than the 19-protein panel(AUC = 0.98), the number of proteins is four could reduce the detecting cost to some extent. Our diagnostic composite panel included four biomarkers corresponding to Aβ transport and ablation (sLRP1 and GSN) [14, 25], cell apoptosis (Caspase 3), as well as the novel potential biological processes (KLK4), which we hope will provide valuable diagnostic strategies for AD, while shedding new light on the pathogenesis and disease progression of AD.

### Limitation

In the study, the cohort analysis was conducted based on clinical diagnosis. The four-blood-based protein panel could classify AD or aMCI from CN with high accuracy. In a plasma proteomic profiling study, the performance of a 19-protein biomarker panel was compared with that of plasma ATN biomarkers, which was superior to that of the integrating model of three plasma ATN biomarkers [10]. As individual proteins responding special biological processes, these biomarkers representing Aβ and tau disorders were required to be measured, when to assess the contributions of four proteins to the pathogenic mechanism and progression of AD. Despite the blood-based protein panel developed to classify AD and MCI with good accuracy, additional testing of the panel was required on other neurodegenerative diseases in the future. As previously reported, plasma sLRP1 was decreased in both AD and vascular dementia (VaD), and unaltered in non-AD neurodegenerative dementia (NND) [21]. However, plasma sLRP1 was much lower in AD than that in VaD [21]. The plasma level of Caspase 3 was unaltered in PSP and Parkinson’s disease [11]. As far as we know, the plasma level of KLK4 was not reported in other neurodegenerative diseases, as well as GSN. Therefore, replication studies examining the blood-based protein panel will be required in a larger cohort, which was including AD and many other neurodegenerative diseases. Finally, given that so many proteins were reported to be altered in the blood in AD by multiplex proteomics in many cohorts [10, 11], it is worthwhile to explore other repeatable and valuable biomarkers, and carefully refine the model by inclusion of other biomarkers, the results of which might help develop a more comprehensive and accurate blood-based screening test.

## Conclusions

Taken together, we measured the plasma levels of four potential biomarkers in AD, aMCI, and CN. We developed an accurate diagnostic composite panel based on the quantification of four biomarkers in plasma for AD and aMCI classification in discovery, validation, and combined cohorts. In addition, we showed that these plasma proteins are remarkably correlated with cognitive assessment among CN, aMCI, and AD. Thus, the blood-based protein panel was established for classifying AD and aMCI, which provides an alternative approach for developing a blood-based test for AD and MCI screening.

## Electronic supplementary material

Below is the link to the electronic supplementary material.

Supplementary Material 1

## Data Availability

The datasets used and/or analyzed during the current study are available from the corresponding author on reasonable request.

## List of Abbreviations

AD: Alzheimer’s disease
aMCI: Amnestic mild cognitive impairment
CN: Normal cognitions
APOE: Apolipoprotein E
sLRP1: Soluble low-density lipoprotein receptor-associated protein 1
GSN: Gelsolin
KLK4: Kallikrein 4
ROC: Receiver operating characteristic
AUC: Area under the curve
PET: Positron emission tomography
CSF: Cerebrospinal fluid
MoCA: Montreal Cognitive Assessment
NIA-AA: National Institute on Aging and Alzheimer’s Association
ELISA: Enzyme-linked immunosorbent assay.
