# Supplementary material for: A blood-based composite panel that screens Alzheimer’s disease
Source: Biomark Res. 2023 May 16;11:53. doi: 10.1186/s40364-023-00485-6 (PMC10189970; doi:10.1186/s40364-023-00485-6)

**Supplementary Information**

| Table S1 The information of ELISA Kits used in this study | | |
| --- | --- | --- |
| Testing Target | Catalog numbers | Manufacturer |
| sLRP1 | SEB010Hu | Cloud-Clone Corp.Wuhan |
| KLK4 | CSB-EL012455HU | CUSABIO |
| Gelsolin(GSN) | SEA372Hu | Cloud-Clone Corp. Wuhan |
| Caspase 3 | ZN2053 | Beijing Biolaibo Technology Co., LTD |

**Figure** **Legends**

Fig. S1. The distribution of four plasma proteins between APOE *ε4* carriers and non-carriers. A-D shows plasma levels of sLRP1, KLK4, GSN, and Caspase 3. Red denotes APOE *ε4* carriers. Blue denotes APOE *ε4* non-carriers. **P* < 0.05, ***P* < 0.01, ****P* < 0.001

Fig. S2. The distribution of four plasma proteins between females and males. A-D shows plasma levels of sLRP1, KLK4, GSN, and Caspase 3. Red denotes female. Blue denotes male. **P* < 0.05, ***P* < 0.01, ****P* < 0.001

Fig. S3 Four blood-based biomarkers and their composite for AD and MCI classification. A, Receiver operating characteristic (ROC) curves showing the performance of four blood-based biomarkers and their composite for AD classification in combined cohorts. B, ROC analyses for MCI classification in combined cohorts

Fig. S1


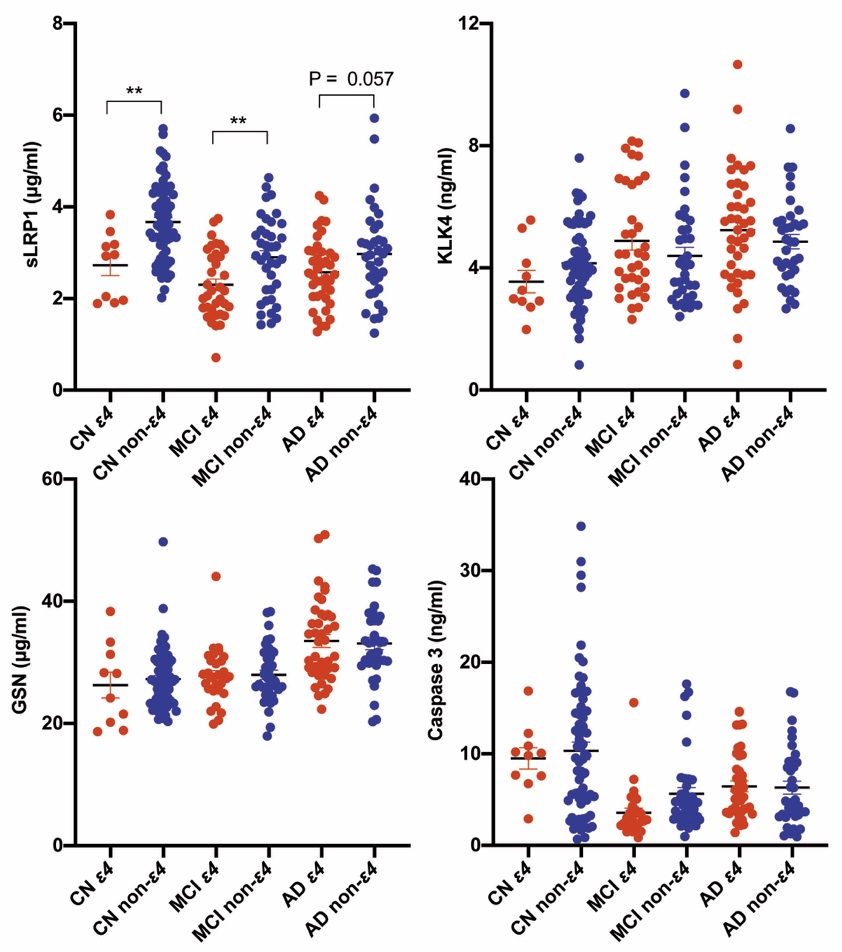


Fig. S2


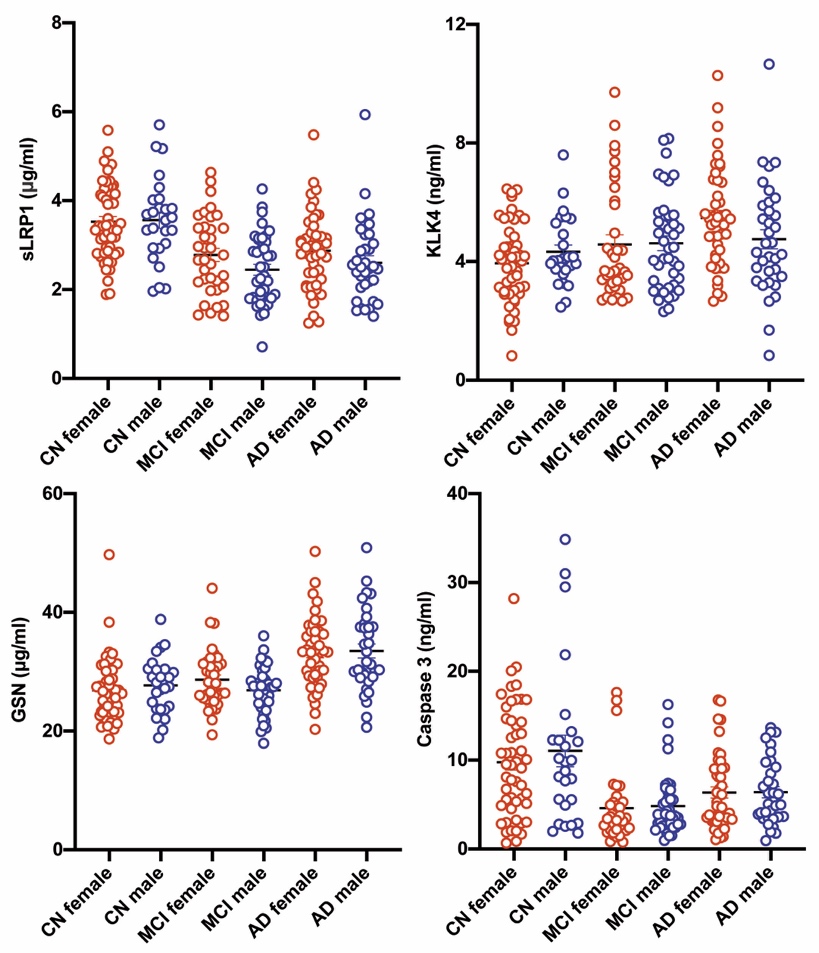


Fig. S3


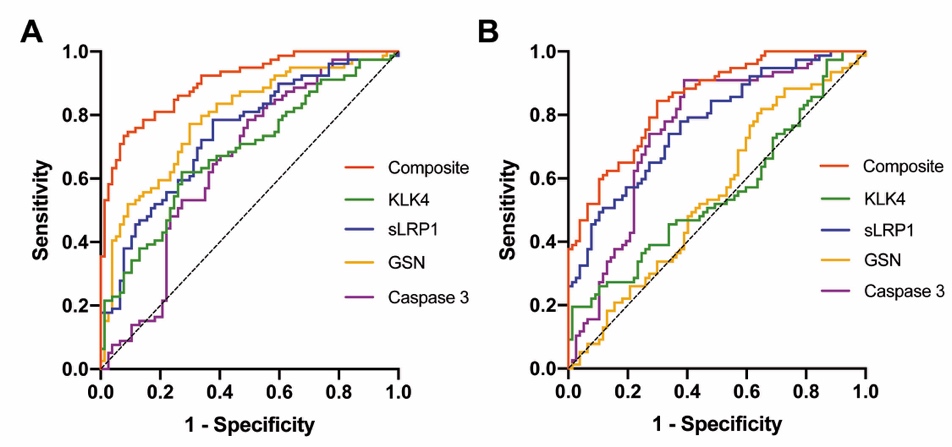

Supplement: Supplementary file 1 — Supplementary Material 1 [file 40364_2023_485_MOESM1_ESM.docx]
